# VCD Studies on Chiral Characters of Metal Complex Oligomers

**DOI:** 10.3390/ijms14010964

**Published:** 2013-01-07

**Authors:** Hisako Sato, Akihiko Yamagishi

**Affiliations:** 1 Department of Chemistry, Graduate School of Science and Engineering, Ehime University, Matsuyama 790-8577, Japan; 2 Department of Chemistry, Toho University, Funabashi 274-8510, Japan; E-Mail: akihiko.yamagishi@sci.toho-u.ac.jp

**Keywords:** chiral, multi-nuclear metal complex, vibrational circular dichroism, chiral induction, β-diketonato, tecton, supramolecule, circular dichroism, DFT calculation, catalysis, low-lying electronic states

## Abstract

The present article reviews the results on the application of vibrational circular dichroism (VCD) spectroscopy to the study of stereochemical properties of chiral metal complexes in solution. The chiral characters reflecting on the vibrational properties of metal complexes are revealed by measurements of a series of β-diketonato complexes with the help of theoretical calculation. Attention is paid to the effects of electronic properties of a central metal ion on vibrational energy levels or low-lying electronic states. The investigation is further extended to the oligomers of β-diketonato complex units. The induction of chiral structures is confirmed by the VCD spectra when chiral inert moieties are connected with labile metal ions. These results have demonstrated how VCD spectroscopy is efficient in revealing the static and dynamic properties of mononuclear and multinuclear chiral metal complexes, which are difficult to clarify by means of other spectroscopes.

## 1. Introduction

Chiral metal complexes have been attracting continuous interest from the fundamental areas of coordination chemistry [1–13]. They constitute an important element in asymmetric catalyses, non-linear optics, chiral sensing and biological probes. Their functions are even more enhanced by coupling the stereochemical properties with the electronic and magnetic ones. Among a variety of chiral elements employed in these attempts, the ΔΛ isomerism of tris(chelated) octahedral complexes have played a unique role as a chiral source [12]. It is characterized by a rigid helical structure with D_3_ symmetry. The helical property is amplified by connecting chiral octahedral motifs to form multinuclear complexes.

In order to investigate the chiral properties of metal complexes, a number of spectroscopic methods are applied, such as X-ray analyses, NMR and electronic circular dichroism (ECD) measurements [3]. These methods help us to obtain the static and dynamic details of chiral metal complexes in solid and solution. As far as our knowledge is concerned, however, relatively little attention has been paid to vibrational circular dichroism spectroscopy as a measure for such purposes.

In recent years, we have been pursuing the possible utility of the vibrational circular dichroism (VCD) spectroscopy to chiral transition metal complexes [14–25]. VCD measures the differential absorption of left *versus* right circularly polarized IR radiation for molecular vibration transitions [26–29]. In comparison to the ECD spectra, the VCD spectra often provide more detailed information as to the stereochemical characters of chiral metal complexes [26–49]. This is because the method possesses the following advantages over other spectroscopes:

1. The VCD spectrum typically contains many well resolved vibrational bands, whereas ECD tends to show fairly broad band contour. This makes VCD assignment considerably more conclusive than ECD.
2. Most of 3N-6 normal vibrations, which are all well resolved as stated above, are obtained from a single measurement and can be utilized to analyze the chiral character in the molecular vibrations of a given molecule.
3. Even a molecule with no electronic absorption in the UV-visible region can be a target. This property is particularly useful for analyzing such a metal complex as having perfluoro groups [16,18,50,51].
4. The DFT modeling of VCD spectra in the ground electronic state is more reliable than for ECD. Moreover the program is commercially available.

The following sections describe how to analyze the chiral structures of metal complexes and their oligomers in solution by making use of the above advantages [14–24].

## 2. VCD Application to Chiral Mononuclear Metal Complexes

### 2.1. Effects of Electronic Properties of Central Metal Ions on Vibrational Energy Levels

The VCD spectroscopy was applied to a series of β-diketonato complexes, [M(III)(acac)_3_] (acac = acetylacetonato; M(III) = Cr(III), Co(III), Ru(III), Rh(III), Ir(III) and Al(III)) and [M(III)(acac)_2_(dbm)] (dbm = dibenzoylmethanato; M(III) = Cr(III), Co (III) and Ru(III)) [14,15]. It was first noticed that the β-diketonato complexes gave very large VCD signals in comparison to the bipyridine, diamine or cyclometalated complexes. The properties made us to obtain the accurate results for a wide range of complexes within short time (less than 10,000 scans). The optical antipodes gave the mirror-imaged spectra in the region of 1700–1000 cm^−1^, confirming the validity and reliability of measurements.

**Chart 1:**
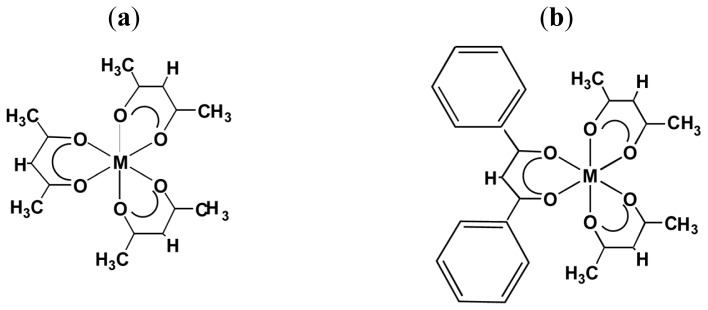
(**a**) [M(III)(acac)_3_] (acac = acetylacetonato, M = Ru, Cr, Co, Rh, Ir and Al); (**b**) [M(III)(acac)_2_(dbm)] (M = Ru, Cr, and Co) (modified from [15]). (**a**) (**b**)

The VCD results showed how the electronic properties of a central metal ion affect the vibrational energy levels and the appearance of low-lying electronic states. The positions of VCD peaks assigned to C–O stretches (1500–1300 cm^−1^) remarkably depend on a central metal ion for both [M(III)(acac)_3_] (Chart 1a) and [M(III)(acac)_2_(dbm)] (Chart 1b). In case of Δ-[M(III)(acac)_3_], for example, the order of frequency of two C–O stretches (E and A_2_ symmetries) is determined as follows: E (negative sign) > A_2_ (positive sign) for M(III) = Co, Rh and Ir, while A_2_ (positive sign) > E (negative sign) for M(III) = Cr and Ru. In case of Δ-[M(III)(acac)_2_(dbm)], the order of frequency of three C–O stretches (A, B and B symmetries) is determined as follows: A (negative sign) > B (positive sign) > B (positive sign) for Co(III), B (positive sign) > A (negative sign) > B (negative sign) for Cr(III) and A (negative sign) > B (positive sign) > B (negative sign) for Ru(III). The results imply that the energy levels of C–O stretches are delicately affected by the electronic configuration of a central metal ion. Since no such detailed information is obtained from the IR spectra alone, the VCD spectrum is a unique method to probe the effect of a central metal ion on interligand cooperative vibrational modes.

It is a general belief that magnetic field perturbation (MFP) theory gives reliable reports on VCD results for the closed-shell systems but not for the open-shell systems. In order to interpret the VCD spectra of paramagnetic metal ions theoretically, the more accurate treatment is required [27,30]. For example, the open-shell density functional theory (DFT) calculations with M06 exchange-correlation functional and all electron Douglas-Kroll second order scalar relativistic correction (DK2) are performed to interpret the vibrational circular dichroism (VCD) spectra of four kinds of tris(acetylacetonato)metal(III), [M(III)(acac)_3_] (acac = acetylacetonato, M = Ru, Cr, Co and Rh) [20]. It is deduced that the experimental spectra are well reproduced by the calculation with harmonic approximation in case of [Co(III)(acac)_3_] (d6; S = 0), [Rh(III)(acac)_3_] (d6; S = 0) and [Ru(III)(acac)_3_] (d5; S = 1/2). In case of [Cr(III)(acac)_3_] (d3; S = 3/2), however, anharmonic effects should be taken into account to predict the accurate vibrational frequencies of closely located modes. We found experimentally that the order of closely locating vibrational energy levels is dependent on a metal ion in case of the same type of metal complex (or [M(acac)_3_]). For example, the relative position of two stretching vibrations of C–O bonds (A_2_ and E symmetries) is opposite for Co(III) and Cr(III) complexes. From the theoretical treatment, this was caused by the anharmonic effects.

Time-dependent density functional theory (TDDFT) calculations are performed to estimate the contribution of excited states in the VCD spectra. As a consequence, the presence of the low-lying excited states is predicted for [Ru(III)(acac)_3_] alone, which agrees with the experimental observation.

These calculations implied that the more accurate treatment is needed in order to take into account the following factors: (i) the anharmonic effects of molecular vibrations, (ii) the contribution of relativistic effects particularly to a complex with a heavy metal ion and (iii) the contribution of low-lying excited states particularly in case of a paramagnetic ion.

### 2.2. Identification of Geometrical and Diastereomeric Isomers of Octahedral Complexes

The VCD spectroscopy is applied to identify the isomers of mixed-ligand Co(III) complexes. The spectra were measured on the chloroform solutions of a series of Co(III) complexes, [Co(tfac)*_n_*(acac)_3−_*_n_*] (*n* = 0 ~ 3, tfac = 1,1,1-trifluoro-2,4-pentanedionato; acac = acetylacetonato) [18]. All of seven pairs of optical antipodes gave mirror-imaged spectra over the whole wavenumber range (1000~1800 cm^−1^), confirming the reliability of measurements. The obtained spectra are compared among the geometrical isomers of bis(tfac) and tris(tfac) complexes. As a result, the difference among the isomers is manifested most remarkably in C–O stretches and C–C–C bending in cooperation with C–CF_3_ stretching. Distinct differences are observed in the VCD spectra among the geometrical isomers of the same ligand composition. Such discrimination is hardly possible by their IR spectra alone.

The structural identification of the diamagnetic Co(III) isomers is performed in conjunction with DFT calculations. In the case of bis(tfac) complexes, the theory is successful in predicting the spectral feature of the band in both C–O stretches and C–C–C bending in cooperation with C–CF_3_ stretching.

**Chart 2:**
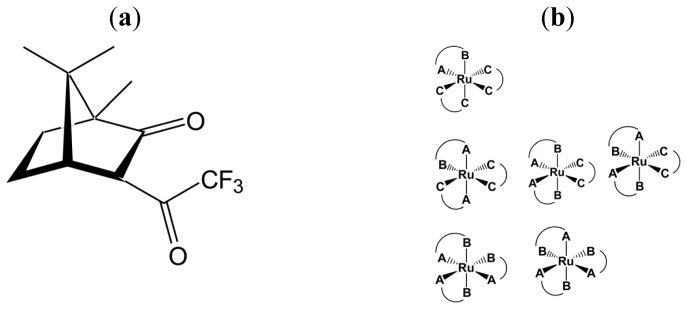
(**a**) (−)-tfac = (−)-3-trifluoroacetylcamphorato); (**b**) Δ- or Λ-[Ru((−)- or (+)-tfac)*_n_*(acac)_3−_*_n_*] (*n* = 1, 2 and 3, (−)- or (+)-tfac = (−)- or (+)-3-trifluoroacetylcamphorato and acac = acetylacetonato ) (modified from [16]).

As another attempt, a complete series of mixed ligand Ru(III) complexes, which are expressed by the formula of Δ- or Λ-[Ru((−)- or (+)-tfac)*_n_*(acac)_3–_*_n_*] (*n* = 1, 2 and 3, (−)- or (+)-tfac = (−)- or (+)-3-trifluoroacetylcamphorato and acac = acetylacetonato) (Chart 2), were prepared in a pure diastereomeric form [16]. The separation of these diastereomers was accomplished chromatographically by rational use of two antipodal chiral columns. The separated complexes were identified by means of mass spectra, ^1^H-NMR, electronic circular dichroism and partly X-ray diffraction analyses. When a pair of Δ- and Λ-[Ru(acac)_3_] (*n* = 0) is added to this group, they constitute *the complete series* of mixed ligand complexes including ligand chirality under the condition of no mixing of chiral ligands.

The VCD spectra of their CDCl_3_ solutions were recorded in the wavenumber region of 1000~1800 cm^−1^. The results provide a benchmark for examining systematically the effects of the stereochemical properties on VCD spectra such as the degree of ligand substitution, ΔΛ configurations, geometrical isomerism and ligand chirality. As a result, the geometrical isomers of trans- or cis-[Ru((−)- or (+)-tfac)_2_(acac)] and mer- or fac-[Ru((−)- or (+)-tfac)_3_] are clearly differentiated by their VCD signals, which is hardly possible from their IR spectra alone. Notably the main spectral features are predicted by the VCD theory based on the magnetic field perturbation (MFP). This is surprising since the present theory is shown to be effective to reproduce the vibrational details even for open-shell metal complexes.

## 3. VCD Application to Oligomers of Chiral Metal Complexes in Solution

### 3.1. Induction of Chiral Structures in Multinuclear Complexes

The VCD spectroscopy is extended to the study of chiral multinuclear complexes in solutions. Heterometallic oligomers were prepared by connecting bis(β-diketonato)ruthenium(III) moieties to a labile metal ion through bis-β-diketonato bridging ligands [19,21,22,52,53]. The main attention is focused on the possibility of the induction of chiral coordination structures around the labile metal ion.

**Chart 3:**
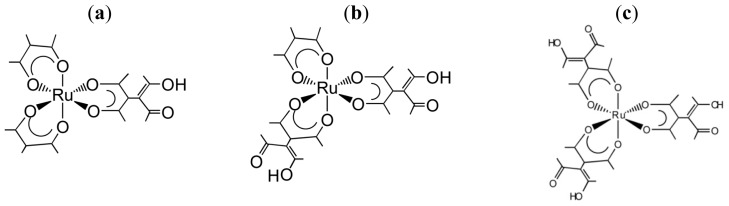
(**a**) one-handed, (**b**) two-handed and (**c**) three-handed tectons (modified from [19,52]).

Complex ligands, [Ru(III)(acac)*_n_*(taetH)_3−_*_n_*] (acacH = acetylacetone, taetH_2_ = tetraacetylethane), are obtained as a chiral form. Using these complexes as a chiral unit, it is attempted to build-up the chiral macromolecules under the control of the number and orientation of connectivity. The approach is denoted as *chiral tectonics*. Accordingly these complexes are denoted as one-handed, two-handed and three-handed tectons for 2, 1 and 0 (Chart 3), respectively.

As the first application of chiral tectons, three units of chiral one-handed tecton, Δ- or Λ- [Ru(acac)_2_(taetH)], are assembled around an aluminum(III) ion to form a star-shaped tetranuclear complex, [{Ru(acac)_2_(taet)}_3_Al(III)] [52]. The remarkable features appear in case of [{Δ-Ru(III)(acac)_2_(taet)}_3_Al(III)]. A fresh chloroform solution of a [{Δ-Ru(III)(acac)_2_(taet)}_3_Al(III)], gives three single distinct peaks due to CH_3_ protons in ^1^H NMR spectra. As time elapses, each single peak begins to split into two composites in about an hour. In addition, the ECD (electronic circular dichroism) spectrum of a freshly prepared chloroform solution of [{Δ-Ru(III)(acac)_2_(taet)}_3_Al(III)] is measured. The spectrum changes in an hour, accompanied by the decrease of the positive peak around 320 nm with the simultaneous increase of the positive peak at 275 nm. The final spectrum is nearly identical to that of Δ-[Ru(III)(acac)_2_(taetH)]. It is concluded that the central labile core of Al(III) in [{Δ-Ru(III)(acac)_2_(taet)}_3_Al(III)] takes initially the coordination structure analogous to Λ-[{Δ-Ru(III)(acac)_2_(taet)}_3_Al(III)] and that it epimerizes slowly to a diastereomeric mixture of Λ- and Δ- [{Δ-Ru(III)(acac)_2_(taet)}_3_Al(III)]. In other words, the coordination sphere around the central Al(III) is locked as a chiral form antipodal to the peripheral parts in the solid state [52]. This example exhibits that the incorporation of a labile metal ion introduces dynamical aspects through rapid ligand substitution processes. An attempt at obtaining the VCD evidence for chiral locking in a solid state is now under progress.

**Chart 4:**
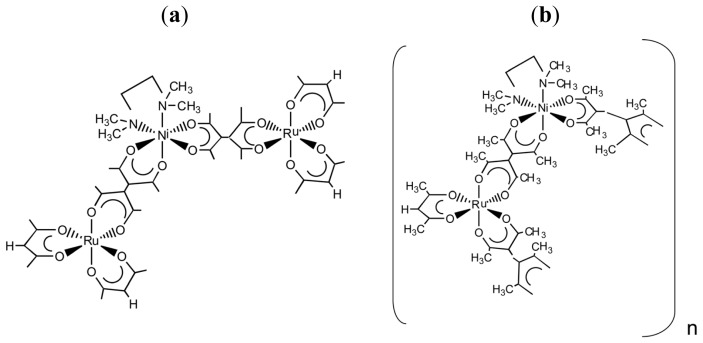
(**a**) [{Ru(III)(acac)_2_(taet)}_2_Ni(tmen)]; (**b**) [{Ru(III)(acac)(taet)_2_Ni(II)(tmen)} *n*{Ni(II)(tmen)}](ClO_4_)_2_ (modified from [19]).

As another application, heterometallic oligomers are formed by reacting one or two-handed chiral tecton, Δ- or Λ-[Ru(III)(acac)*_n_*(taetH)_3−_*_n_*] (*n* = 2 or 1, respectively), with labile Ni(II) ions in a solution [19]. These chiral tectons react with Ni^2+^ ions in the presence of excess *N*,*N*,*N′*,*N′*- tetramethylethylenediamine (tmen) in methanol or methanol/chloroform. In case of one-handed tecton, a trinuclear complex, [{Ru(III)(acac)_2_(taet)}_2_Ni(II)(tmen)] (Chart 4a), is formed at the ratio of Ni(II)/Ru(III) = 0.5. No chirality is induced in the coordination spheres of labile Ni(II) connectors.

In case of two-handed tecton, a soluble oligomer, [{Ru(III)(acac)(taet)_2_Ni(II)(tmen)}*_n_*Ni(II)(tmen)]^2+^ (*n*~4) (Chart 4b), is produced around Ni(II)/Ru(III) = 1.0. The ECD and VCD spectra are measured in order to examine the possibility of chiral induction in the produced oligomers. When the reaction is monitored by ECD, a new peak appears in the region of 300~400 nm, where the portion of –[(taet)Ni(II)(tmen)(taet)]-in the oligomers has an electronic absorption. The sign of the peak reverses, depending on the ΔΛ configuration of the two-handed tecton. Since the peaks exist in the wavelength region corresponding to the electronic absorption bands due to [Ni(acac)_2_tmen], it is suggested that the peak is caused by the chiral octahedral coordination around Ni(II) ion. Since no CD spectrum is reported for [Ni(acac)_2_tmen] because of its labile properties, it is unknown which of the absolute configuration, Δ or Λ, the Ni(II) ions take in the oligomers.

The VCD spectra of a 4:1 (*v*/*v*) CD_3_Cl/CD_3_OD solution containing chiral [Ru(III)(acac)(taetH)_2_], Ni(ClO_4_)_2_ and tmen at a ratio of 1:1:10 were measured. The spectra correspond to either {Δ-[{Ru(III)(acac)(taet)_2_Ni(II)(tmen)}*n*{Ni(II)(tmen)}](ClO_4_)_2_ or {Λ-[{Ru(III)(acac)(taet)_2_Ni(II) (tmen)}_n_{Ni(II)(tmen)}](ClO_4_)_2_. The new VCD signal is observed around 1250 cm^−1^, where the chiral two-handed tectons show no absorption peak. The sign of the peak inverses, depending on the used Λ- and Δ-two-handed tectons. In order to make clear the origin of the new peak, the VCD spectrum of Λ-[Ni(acac)_2_(tmen)] is calculated theoretically. Comparing the observed results with the calculated ones, the positive and negative peaks around 1250 cm^−1^ in the observed spectra are concluded to be due to Λ- and Δ-configurations around the Ni(II) ions, respectively. In other words, the configuration around the Ni(II) ions in the oligomers is opposite to that of the chiral Ru(III) tectons.

It should be emphasized that such induction of chirality is possible only when a labile Ni(II) ion is connected with the inert two-handed but not with one-handed Ru(III) ions. The results imply that the steric control around the Ni(II) ion in the two-handed way is necessary to stabilize its chiral structure. The VCD spectroscopy is useful in analyzing these chiral oligomeric species in solution.

### 3.2. Chiral Structures of Microdomains in Multinuclear Complexes

The VCD spectroscopy enables us to characterize the nature of the microdomains in supramolecules. In order to exemplify such application, a star-burst type tetranuclear complexes, Δ-(or Λ-)[{Δ-(or Λ-)Ru(III)(acac)_2_(taet)}_3_Ru(III)], was synthesized by reacting three-handed tecton (Chart 3c) with bis-chelated Ru(III) units [53]. As a result, three bis(acetylacetonato)Ru(III) moieties are connected with one tris(chelated)Ru(III) core.

**Chart 5:**
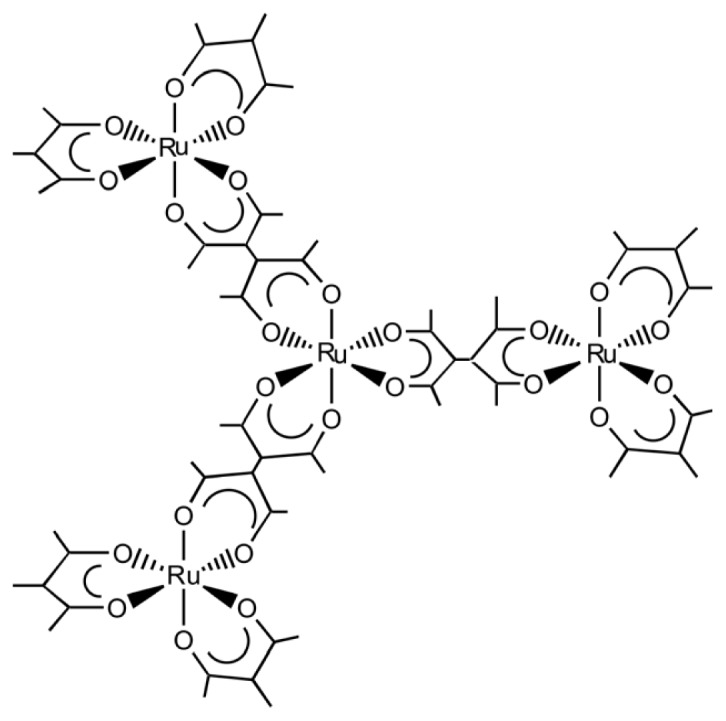
Star-burst type tetranuclear complexes, Δ-(or Λ-)[{Δ-(or Λ-)Ru(III)(acac)_2_ (taet)}_3_Ru(III)] from three-handed tecton (modified from [22]).

The molecule is characterized by the dual structure consisting of the central core ((taet)_3_Ru(III)) and the peripheral region ({Ru(III)(acac)_2_}_3_). The net chirality due to the ΔΛ isomerism of four chiral Ru(III) moieties is manifested in the peak intensity of ECD spectra. It remains to be clarified, however, how to characterize spectroscopically the homo- or hetero-connectivity between the tris(chelated) central core and the bis(chelated) peripheral moieties. VCD spectroscopy is applied to study the stereochemical properties of a star-burst type tetranuclear Ru(III) complex, Δ- (or Λ-)[{Δ- (or Λ-)- Ru(III)(acac)_2_(taet)}_3_Ru(III)] (acac = acetylacetonato; taet = tetraacetylethanato) (Chart 5) [22].

**Scheme 1:**
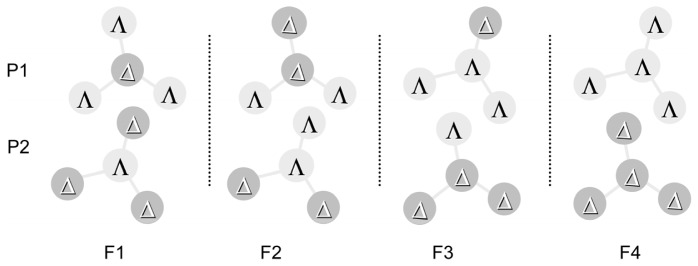
Four possible enantiomeric pairs represented by (F1) Δ–ΛΛΛ/Λ–ΔΔΔ F2 Δ–ΛΛΔ/Λ–ΔΔΛ F3 Λ–ΛΛΔ/Δ–ΔΔΛ and (F4 Λ–ΛΛΛ/Δ–ΔΔΔ, respectively (modified from [22]).

By comparing the VCD spectra of eight diastereomeric enantiomers (Scheme 1), it is shown that the spectra reflect the homo- or hetero-chiral nature of the bridging part connecting the central core and the peripheral region. The sign of the band of 1500~1550 cm^−1^ is positive for P1 and negative for P2 for all diastereomers. In other words, the band has the positive sign for the Λ-rich peripheries, while it has the negative sign for the Δ-rich peripheries. In this sense, the peak around 1340 cm^−1^ reflects the net chiral characters of the three peripheral moieties.

**Figure 1:**
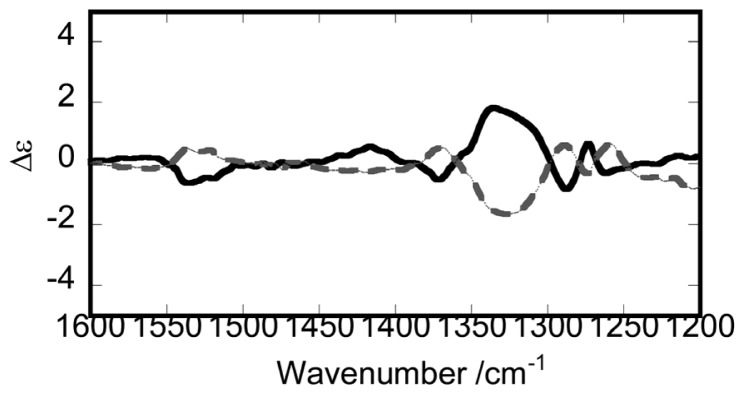
The vibrational circular dichroism spectra of the optically resolved enantiomers of F2 in CDCl_3_. Solid and dotted lines correspond to P1 and P2 as shown in Scheme 1, respectively, (modified from [22]).

The VCD spectra of F2 enantiomers exhibits the intense peak around 1340 cm^−1^ as shown in Figure 1. This is interesting because the F2-type enantiomers possess two Λ- and two Δ-Ru(III) elements, resulting in the cancellation of the net ΔΛ isomerism. In fact, its ECD spectrum (Δɛ) is very low in comparison to other diastereomers. The expression means that Δɛ was small because of the cancelling out of the main ΔΛ chirality, leaving only the minor chirality due to the chirality of a bridging part. Accordingly the observed high intensity in VCD spectra is suspected to reflect the local chirality in the molecules. The band at 1340 cm^−1^ is assigned to the C–C–C stretches of acetylacetonato groups.

### 3.3. Axial Chirality in a Dimer Bridged with Non-Symmetric Bis(β-diketonato)

The multi-nuclear complexes appearing in the previous section possess Δ- or Λ-tris-β-diketonato parts as a chiral unit, while the bridging parts are achiral since they are connected by symmetric bis(β-diketonato) ligand with D_2d_ symmetry. In this section, we report the chiral induction of a bridging part by use of an non-symmetrical β-diketonato ligand, in which the twisting of two β-diketonato planes causes C_2_ symmetry [21,24].

**Scheme 2:**
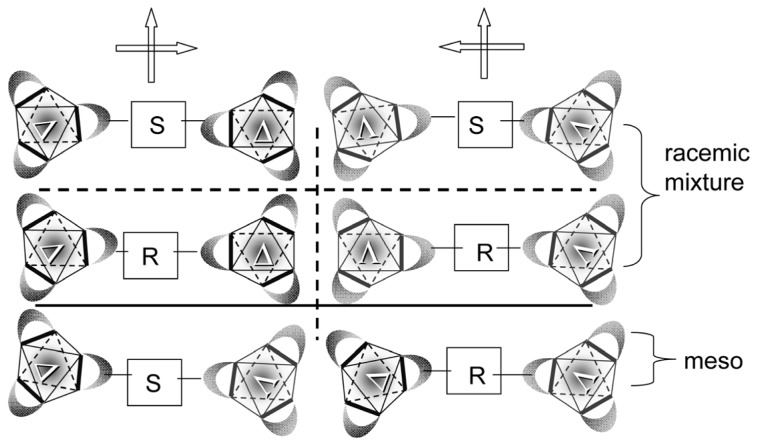
ΔΔ- (or ΛΛ-)[Ru(III)(acac)_2_(*S*-dabe or *R*-dabe)Ru(III)(acac)_2_] or ΔΔ- (or ΛΛ-)[Ru(III)(acac)_2_(*R*-dabe or *S*-dabe)Ru(III)(acac)_2_] (racemic type) and ΔΛ-[Ru(III)(acac)_2_(*S*-dabe or *R*-dabe)Ru(III)(acac)_2_] (meso-type) with non-symmetrical orthogonal bridging ligands.

**Chart 6:**
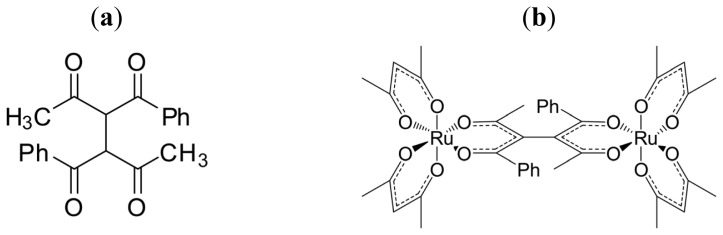
Structures of (**a**) 1,2-diacetyl-1,2-dibenzoylethane (dabeH_2_) and (**b**) [Ru(III)(acac)_2_(dabe)Ru(III)(acac)_2_] (modified from [21]).

As the precursor of such a ligand, 1,2-diacetyl-1,2-dibenzoylethane (dabeH_2_), is used (Chart 6a). The meso-type and racemic diastereomers of dinuclear complex, ΔΛ- and ΔΔ- (or ΛΛ)-[Ru(III)(acac)_2_(dabe)Ru(III)(acac)_2_] (Chart 6b), are obtained. From the ^1^H-NMR spectra of these complexes in CDCl_3_, the two tris-β-diketonato moieties in the bridging part are unequivalent for the meso-type dimer, while they are equivalent for the racemic dimers as shown in Scheme 2. The results are rationalized by assuming that the bridging part (dabe^2−^) is chiral (denoted as *S*-dabe and *R*-dabe tentatively). Based on this assumption, the meso-type dimer is assigned to ΔΛ-[Ru(III)(acac)_2_(*S*-dabe or *R*-dabe)Ru(III)(acac)_2_] (meso-type), while the racemic dimers are assigned to either ΔΔ- (or ΛΛ-)[Ru(III)(acac)_2_(*S*-dabe or *R*-dabe)Ru(III)(acac)_2_] or ΔΔ- (or ΛΛ-)[Ru(III)(acac)_2_(*R*-dabe or *S*-dabe)Ru(III)(acac)_2_] (racemic type). When methanol solutions of these dimers are eluted on a chiral column, the more and less retained fractions give mirror-imaged ECD spectra, indicating that the meso-type dimer consists of a pair of optical antipodes. It should be noted that the resolved enantiomers of the meso-type dimer give much weaker CD peaks than the racemic dimers. This is reasonable, since the chirality due to the ΔΛ isomerism of two tris-β-diketonato parts are cancelled out, leaving the chirality of the bridging part alone for the meso-type dimer.

**Figure 2:**
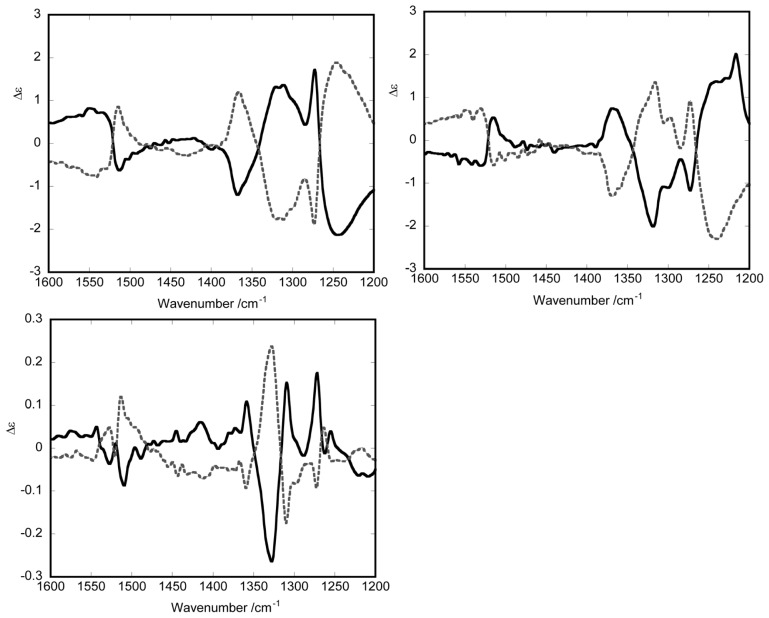
The vibrational circular dichroism spectra of the optically resolved enantiomers of racemic types (upper left and right) and meso-type (under) in CDCl_3_. Solid and dotted lines correspond to less and more retained enantiomers, respectively. It should be noted that the peak intensity for the meso-type dimers is ten times lower than that for the racemic dimers (modified from [21]).

When the VCD spectra are measured on these dimers as a CDCl_3_ solution, the intensities of VCD peaks are also much lower for the meso-type dimer than for the racemic dimers as shown in Figure 2. In the case of the meso-type dimer, the triple multiple peaks with different signs are observed with lower intensity around 1550 cm^−1^. The appearance of these peaks might come from the stretching vibration of C–O bonds in the bridging part. The multiple peaks in the region of 1300–1400 cm^−1^ give a clue to determining the *RS* configuration of the bridging part. More detailed theoretical analyses to determine the absolute configuration from the VCD spectra are now in progress.

## 4. Future Development

### 4.1. Monitoring the Structural Change of Chiral Aggregates by Means of VCD

In the previous sections, VCD spectroscopy is applied to study the structure and vibrational properties of a chiral molecule that is dissolved in a solution with no structural change. In contrast to those static applications, another possibility is that the spectroscopy is used to study the structural transformation or chemical reactions involving chiral molecules in a solution (or dynamic applications). For that purpose, the VCD spectra are measured as a function of time during chemical reactions or by changing the external parameters such as temperature or the concentration of reactants.

One example of applying VCD spectroscopy to the structural change of chiral systems is to follow the photo-induced change of chiral liquid crystals [13,25]. In this example, the real time monitoring of helical rewind process in chiral nematics liquid crystals is performed by use of VCD spectroscopy. As a result, it is revealed that the rewinding of supramolecular helices is monitored *in situ* in a chiral nematic phase under the illumination of UV light (365 nm). Here the change is caused by the photoracemization of a doped chromium(III) complex. It should be noted that the rewinding process of the helix is visualized as the change of the spectral shape at each of the four vibrational peaks. This is the first application of VCD spectroscopy to dynamical systems.

### 4.2. VCD Application to Asymmetric Reactions in Solutions

As another example of dynamical applications, the VCD spectra were measured as a function of time for the asymmetric reactions involving chiral metal complexes. A nickel(II) complex with (*R*)- or (*S*)-2,2′-bis(salicylideneamino)-1,1′-binaphthyl was synthesized and its crystal structure was determined. In a solid, the nickel complex forms a binuclear species consisting of two Ni(II) ions and homochiral two ligand dianions. Each Ni(II) ion has trans-N_2_O_2_ square-planar coordination sphere. In a solution, the same complex transforms its coordination structures among square-planar, tetrahedral and octahedral ones, depending on solvent and/or temperature [54]. The complex is possible to catalyze the asymmetric syntheses such as epoxidation [55]. On these systems, it is now attempted to apply the VCD spectroscopy *in situ* to follow each step in achieving the discrimination and generation of chirality.

## 5. Summary

In the present paper, the results of our recent application of the vibrational circular dichroism (VCD) spectroscopy to investigate the stereochemical properties of chiral metal complexes in a solution. Attention has mainly been paid to the following aspects of mononuclear and multinuclear metal complexes: (1) the effects of electronic configuration of a central metal ion on the vibrational properties, (2) the identification of geometric and diastereomeric isomers of metal complexes, (3) the chiral structures induced on linking homo- or hetero-metallic ions, (4) the chiral characters of micro-domains in multi-nuclear metal complexes and lastly (5) the future development of the VCD method to the dynamical processes involving chiral metal complexes.
